# Neural Differentiation in HDAC1-Depleted Cells Is Accompanied by Coilin Downregulation and the Accumulation of Cajal Bodies in Nucleoli

**DOI:** 10.1155/2017/1021240

**Published:** 2017-02-27

**Authors:** Jana Krejčí, Soňa Legartová, Eva Bártová

**Affiliations:** Institute of Biophysics of the Czech Academy of Sciences, v.v.i., Královopolská 135, 612 65 Brno, Czech Republic

## Abstract

Cajal bodies (CBs) are important compartments containing accumulated proteins that preferentially regulate RNA-related nuclear events, including splicing. Here, we studied the nuclear distribution pattern of CBs in neurogenesis. In adult brains, coilin was present at a high density, but CB formation was absent in the nuclei of the choroid plexus of the lateral ventricles. Cells of the adult hippocampus were characterized by a crescent-like morphology of coilin protein. We additionally observed a 70 kDa splice variant of coilin in adult mouse brains, which was different to embryonic brains and mouse pluripotent embryonic stem cells (mESCs), characterized by the 80 kDa standard variant of coilin. Here, we also showed that depletion of coilin is induced during neural differentiation and HDAC1 deficiency in mESCs caused coilin accumulation inside the fibrillarin-positive region of the nucleoli. A similar distribution pattern was observed in adult brain hippocampi, characterized by lower levels of both coilin and HDAC1. In summary, we observed that neural differentiation and HDAC1 deficiency lead to coilin depletion and coilin accumulation in body-like structures inside the nucleoli.

## 1. Introduction

Cajal bodies (CBs) are striking nuclear structures consisting of accumulated proteins that play various roles in nuclear processes. These structures were designated Cajal's accessory bodies* (cuerpo accesorio)* and were discovered for the first time in rat brain neurons [1]. A role of the CBs during neurogenesis was also significantly studied and summarized by Lafarga et al. [2] and Baltanás et al. [3]. At this moment, it is well known that the function of these structures is dynamic because CBs regulate RNA synthesis and the assembly of ribonucleoproteins (RNPs) [4]. Moreover, Tapia et al. [5] showed that the symmetrical dimethylation of arginines on coilin supports the formation of CBs, positive on survival motor neuron (SMN) proteins and small nuclear ribonucleoproteins (snRNPs). These regulatory factors probably determine the association of CBs with the spliceosome and a role for CBs in pre-mRNA splicing [6]. Conversely, coilin hypomethylation depreciates its function and causes the disintegration of canonical CBs into small microfoci. Unmethylated coilin does not support the formation of robust CBs but is located inside the dense fibrillar component of the nucleoli. In this form, there is no link between the coilin nuclear pattern and global transcription activity [5]. On the other hand, canonical CBs, which are nonmembrane nuclear components, are prominent structures in dividing cells with high transcriptional activity [4]. CBs have a diameter of 0.5–1.0 *μ*m and contain many proteins, including the abovementioned p80 coilin, which becomes increasingly more phosphorylated during mitosis and, particularly in human embryonic stem cells, is present at high levels in the nucleoplasmic pool [7–12]. However, coilin is not completely essential because knockout of coilin in mice is not lethal [13]. On the other hand, coilin-positive CBs play an important role in genome organization in terms of gene expression and pre-mRNA splicing via their association with many chromosomes. The periphery of these chromosomes represents a site of interaction for genes that are poised for transcription and thus associates with regulatory components. Human chromosome 1 is a key player in these processes, and its periphery is frequently occupied by CBs [14]. The use of chromosome conformation capture analysis (4C-seq), a novel molecular biology method, has revealed an association between highly expressed histone genes, sn/snoRNA coding loci, and CBs, which are involved in intra- and interchromosomal clusters [14, 15]. This interaction is of immense functional importance during transcription and especially splicing. CBs are also highly mobile structures, as revealed by single-particle tracking analysis and fluorescence recovery after photobleaching (FRAP) [10, 16–19]. For example, we recently demonstrated the constrained local motion of individual CBs after cell exposure to *γ*-radiation. Furthermore, in mouse embryonic stem cells (mESCs), the coilin dispersed in the nucleoli and accumulated in CBs was characterized by a reduced mobile fraction compared to the GFP-tagged coilin in the nucleoplasm [10]. FRET (fluorescence resonance energy transfer) analysis additionally revealed a specific interaction between coilin and SMN protein in CBs as well as the appearance of coilin-coilin dimerization [17]. However, as regards to DNA repair machinery, our experiments did not show coilin-SMN interaction or coilin dimerization in UVA-induced DNA lesions, which are characterized by pronounced coilin recruitment [10]. Together, the abovementioned results illustrate the dynamic behavior of coilin and CBs, which is required not only for optimal pre-mRNA processing but also for DNA repair [15].

Interestingly, in some tumor cells, the functional properties of coilin are associated with both CBs and nucleoli. The nucleoli contain many different proteins that play a role during the transcription of ribosomal genes or during DNA repair [18–20]. In UVA-damaged chromatin, we observed the recruitment of the upstream binding factor UBF, a major transcription factor for ribosomal genes, and we noted a similar response for coilin [10, 21]. As determined by Boulon et al. [22], UVA and UVC cause the disintegration of coilin-positive CBs, and ionizing irradiation has a similar, notable effect of CB disruption [23, 24]. Thus, nucleolar proteins, including coilin that also appears in nucleoli of tumor cells, appear to be involved in the DNA repair machinery, which is, for example, also activated in Purkinje cells during neurodegeneration, characterized by the disintegration of nucleoli and CBs [3].

In this study, we focused especially on the nuclear distribution patterns of the CBs, and we studied coilin levels in embryonic and adult mouse brains and during neural differentiation of mESCs. Based on the initial observations of Raymond Cajal, who noted that the CBs are striking nuclear components of the rat brain and, more specifically, the pyramidal cells of the human cerebral cortex [1, 25], we analyzed the nuclear distribution patterns and formation of the CBs in the hippocampus and olfactory bulbs (OBs) of adult mouse brains. We also investigated the distribution of coilin in the ventricular ependyma of e15.5 embryonic brains. Furthermore, to elucidate the CB dynamics in neurogenesis, we analyzed the formation of CBs during the neural differentiation of wild-type (wt) and HDAC1 double-knockout (dn) mESCs. From the view of neural differentiation, it was shown that embryonic neural progenitor stem cells are characterized by a high level of HDAC1, while HDAC2 is expressed during neural differentiation and pronouncedly in terminally differentiated neurons [26]. Differentiation processes in the brain are also regulated by HDAC3, as shown by Volmar and Wahlestedt [27]. Moreover, in neural progenitor stem cells, functional HDAC3 was found to be responsible for the balance between cell proliferation and differentiation [28]. Based on these data we addressed the following hypothesis: whether neural differentiation and HDAC1 depletion can affect the levels of coilin and the nuclear distribution of Cajal bodies because we expected that depletion of some HDAC induces chromatin relaxation; thus this nuclear event could change distribution pattern of CBs. We also analyzed HDAC1 depletion in order to show how changes in histone acetylation, a central epigenetic factor responsible for chromatin accessibility [29, 30], can change the level of coilin, which is methylated when it accumulates in CBs [5].

## 2. Results

### 2.1. The Nuclear Distribution Pattern of Cajal Bodies in the Embryonic and Adult Mouse Brain

**Figure 1:**
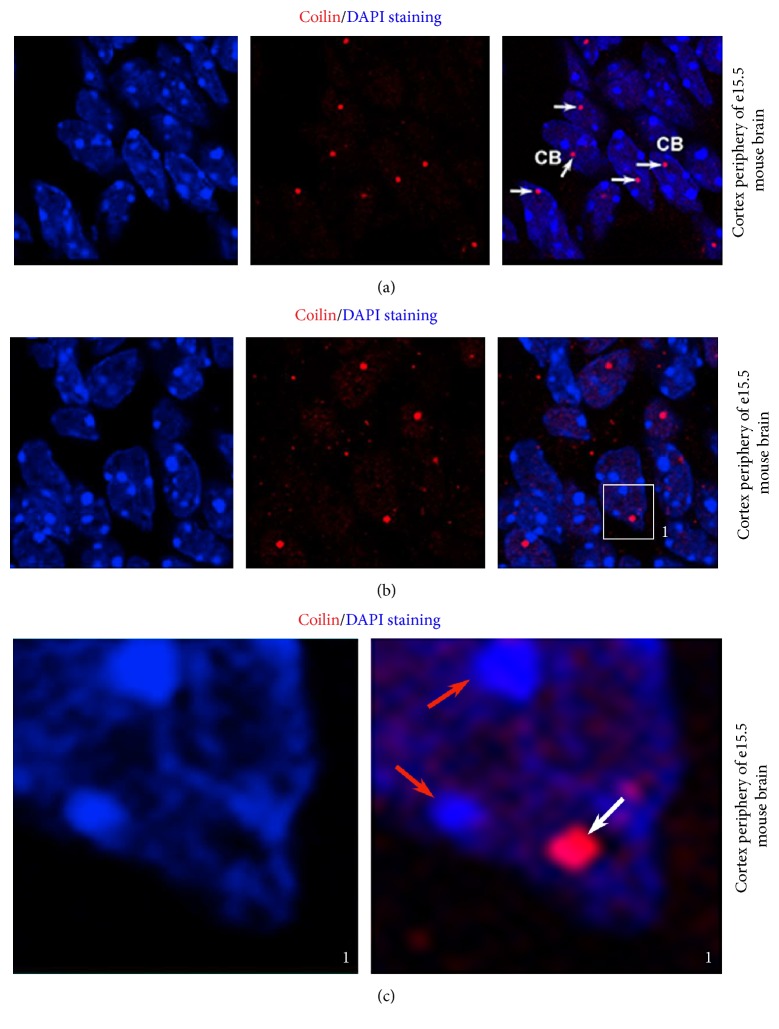
Formation of Cajal bodies (CBs) in the cortex periphery of e15.5 mouse embryonic brains (a–c). CBs were visualized with Alexa 594 fluorescence (red), and DAPI (4′,6-diamidino-2-phenylindole) was used as a counterstain (blue). Arrows in (a) show individual CBs and the frame in (b) shows a selected region in the cell nucleus magnified in (c). Red arrows in (c) indicate the clusters of centromeric heterochromatin (chromocenters) and white arrows show the selected CB.

We inspected sections of embryonic and adult mouse brains and observed the formation of single, robust CBs at the cortex periphery in embryonic brains at stage e15.5 after fertilization (Figures 1(a)–1(c)). We additionally found that in approximately 90% of the cell nuclei at the cortex periphery, the Cajal bodies (CBs) were located away from clusters of centromeric heterochromatin called chromocenters (Figure 1(c)).

**Figure 2:**
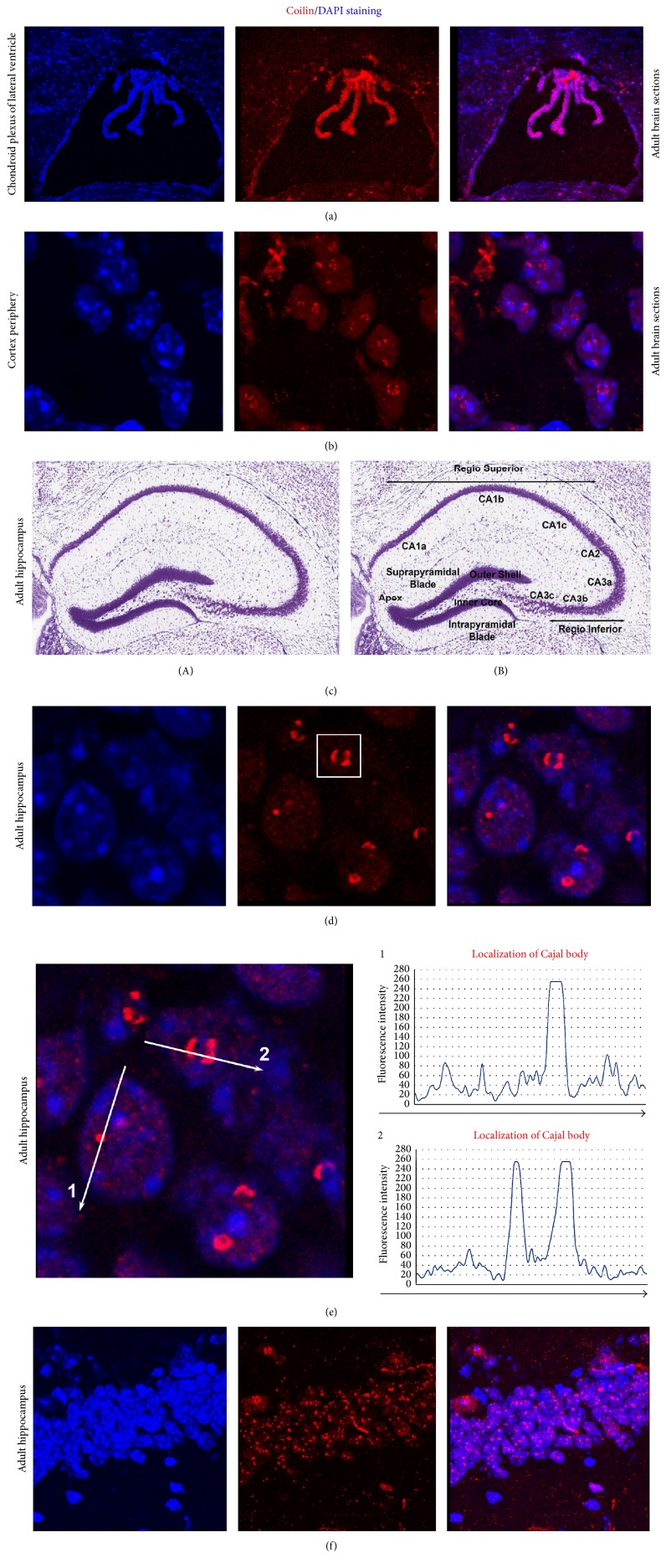
The nuclear distribution patterns of coilin in adult mouse brain sections. (a) shows the chondroid plexus of the lateral ventricle. (b) Coilin distribution in the cortex periphery of an adult brain. ((c)(A), (c)(B)) Hippocampal regions visualized by hematoxylin-eosin staining, an image from the brain atlas (see [31]). (d–f) Accumulation of coilin in crescent-like foci in the hippocampal region of an adult brain. DAPI staining is used to visualize cell nuclei. Coilin (red) was labeled by a secondary antibody conjugated with Alexa 594. Nuclear distribution of coilin in cells 1 and 2 (e) is shown in graphs 1 and 2. Fluorescence intensity along white lines with arrows was measured using the Image J software (NIH freeware). (f) shows a high density of coilin in the hippocampus (hippocampal blade) of an adult mouse brain.

**Figure 3:**
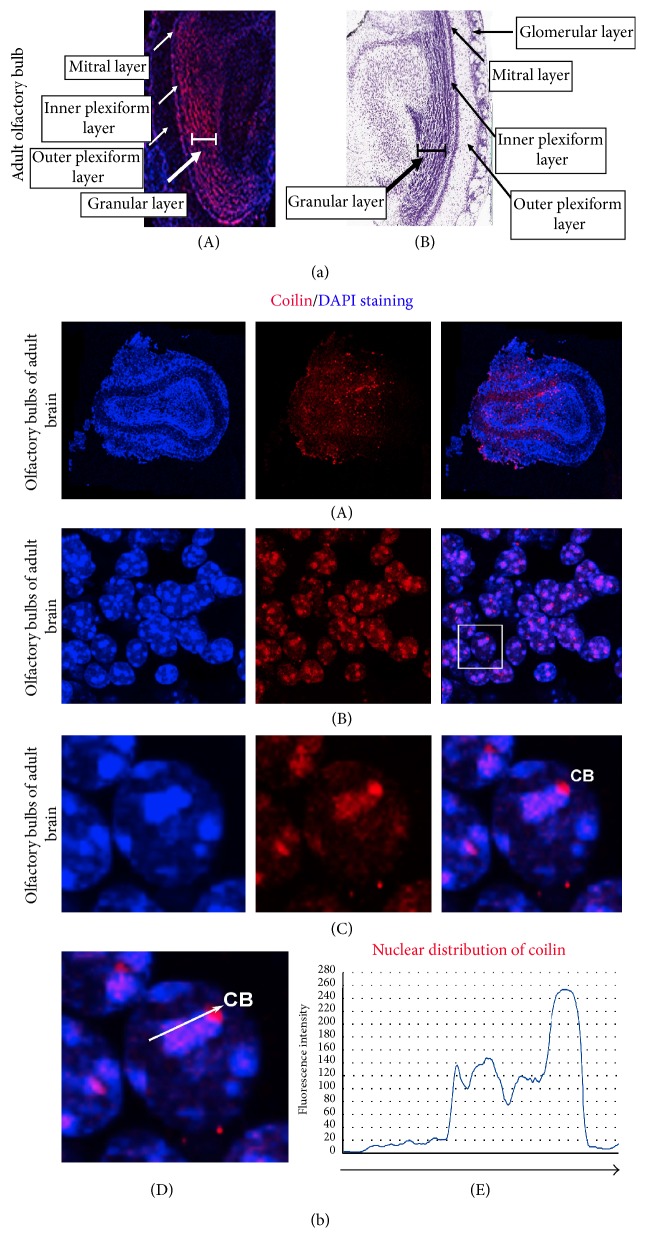
Coilin expression in the olfactory bulbs (OBs) of the adult brain. ((a)(A)) The OB regions of an adult mouse brain visualized by DAPI staining (blue) and an antibody against acetylated histone H3 (red; an antibody raised against H3K9ac [#06-942, Merck Millipore] was used to visualize the granular layer of OB due to its high density). The morphology of the OB in ((a)(A)) is compared with the morphology of the OB according to ((a)(B)) the brain atlas (see [31]). ((b)(A)–(b)(D)) show coilin accumulation in the Cajal bodies. In adult OBs, CBs were surrounded by DAPI- and coilin-dense regions (red and blue) (see (b)(C)). ((b)(D)) shows a magnification of the cell nucleus from OB. ((b)(E)) indicates the density of coilin, visualized by Alexa 594 fluorescence, analyzed across the selected region delineated by a white arrow in ((b)(D)).

The cell nuclei in adult brains were highly positive for the coilin protein, particularly in the chondroid plexus of the lateral ventricle (Figure 2(a)). However, the cells in this region did not have easily discernable CBs. Next, we observed clustering of coilin inside the cell nuclei occupying the cortex periphery in adult mouse brains (Figure 2(b)). Analysis of the hippocampal blade (Figures 2(c)(A) and 2(c)(B)) revealed both the crescent-like accumulation of coilin and individual canonical CBs (Figures 2(d)–2(f)). Surprisingly, in olfactory bulbs (Figures 3(a)(A), 3(a)(B), and 3(b)(A)), high levels of coilin were noted in the highly DAPI-dense nuclear regions surrounding single CBs (Figures 3(b)(B)–3(b)(D)). This nuclear distribution pattern of coilin was observed in individual nuclei of the granular layer of the OBs in adult brain (Figures 3(b)(A)–3(b)(C); see magnification in Figure 3(b)(D) and quantification in Figure 3(b)(E)).

### 2.2. Levels and Nuclear Distribution Pattern of Coilin, Fibrillarin, and SC35 in Mouse Brain and Pluripotent or Differentiated mESCs

**Figure 4:**
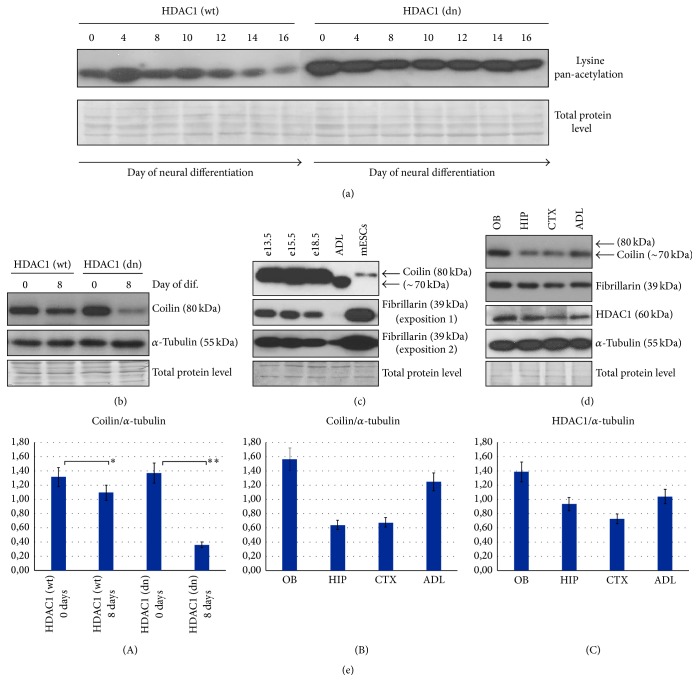
The levels of coilin, HDAC1, and fibrillarin in pluripotent and differentiated mouse ESCs and in the mouse brain. (a) In comparison to nondifferentiated and differentiated wt mESCs, a very high level of lysine pan-acetylation was found in HDAC1 dn cells and in their differentiated counterpart. Neural differentiation was induced in both wt and HDAC1 dn cells by identical differentiation protocol. (b) Western blot shows coilin and *α*-tubulin (reference protein) levels in nondifferentiated and differentiated (neuronal pathway) wt and HDAC1 dn mouse ESCs. HDAC1 depletion in these cells was first published by Lagger et al. [32]. (c) Western blot analysis of the coilin and fibrillarin levels in embryonic mouse brains at developmental stages e13.5, e15.5, and e18.5 and in the whole adult brain as well as in mESCs. Two expositions for fibrillarin were used in order to show the differences between the levels of fibrillarin in the adult mouse brain (ADL) and mESCs. (d) The levels of coilin, fibrillarin, HDAC1, and *α*-tubulin in the following regions of adult brain: the olfactory bulb (OB), the adult hippocampus (HIP), the brain cortex (CTX), and the whole adult mouse brain (ADL). (b–d) show the conclusions from three independent experiments, and the total loaded protein levels are also documented. (e)(A) Quantification of the results from (b); (B) quantification of (c); and (C) analysis of the HDAC1 level from (d). Asterisk (*∗*) denotes statistically significant results at *p* ≤ 0.05 and (*∗∗*) at *p* ≤ 0.01.

In comparison to nondifferentiated and differentiated wt mESCs, pan-acetylation of lysines was very high in HDAC1 dn mESCs and their differentiated counterpart (Figure 4(a)). In these experiments, we addressed a question if hyperacetylated surroundings of CBs in HDAC1 dn mESCs could change formation or maintenance of CBs, which is regulated by methylation-related processes [5].

**Figure 5:**
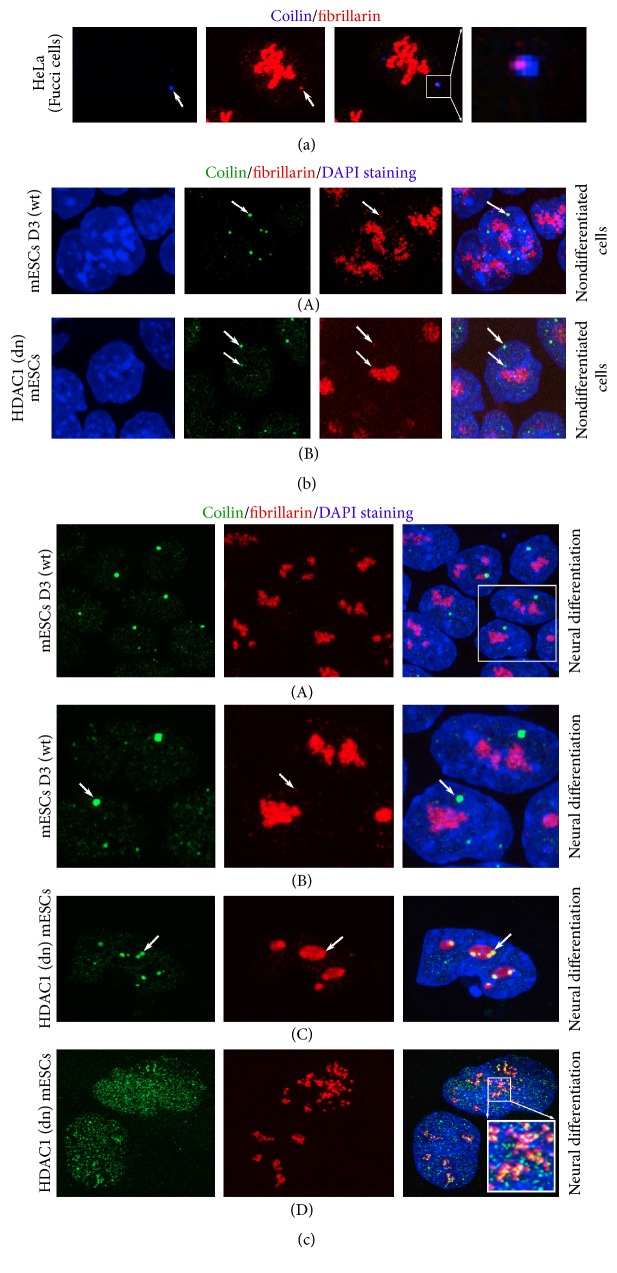
The spatial link between coilin and fibrillarin in HeLa cells and mouse pluripotent mESCs before and after neural differentiation. Arrows show fibrillarin and coilin occurrence in CBs in (a) HeLa cells; CB (blue) colocalizes with fibrillarin foci (red) (white arrows). (b) In (A) wt and (B) HDAC1 dn pluripotent ESCs, CBs (green) were located in a close proximity to the periphery of the fibrillarin-positive regions of the nucleoli (red). An example of CBs is shown by arrows. (c) The spatial link between CBs (green) and fibrillarin (red) in (A, B) wt mESCs and (C, D) HDAC1 dn mESCs undergoing neural differentiation (white arrows). Accumulated coilin (green) inside the nucleoli (red) was observed in HDAC1 dn cells (see (C) and (D)). DAPI staining (blue) was used to visualize the cell nuclei.

Here, western blot analysis revealed reduced levels of coilin (80 kDa) during neural differentiation of wt mESCs (Figures 4(b) and 4(e)(A)). We also analyzed the levels of coilin in nondifferentiated and differentiated wt and HDAC1 dn mES cells. Our statistical analysis, using Student's *t*-test, documented significant changes at ^*∗*^*p* ≤ 0.05 when we compared nondifferentiated and differentiated wt mESCs (Figures 4(b) and 4(e)(A)). In HDAC1-depleted cells, the difference was even more pronounced: a significantly different result (at ^*∗∗*^*p* ≤ 0.01) was found when we compared nondifferentiated and differentiated HDAC1 dn cells (Figures 4(b) and 4(e)(A)). We also examined the coilin levels in mouse brains at various developmental stages. We studied the whole brains of e13.5, e15.5, and e18.5 embryonic stages and adult mice (Figure 4(c)). Compared to embryonic brains, which are characterized by the 80 kDa coilin variant, we observed a different splice variant of coilin (~70 kDa) in adult brains. During mouse brain development, coilin levels were stable at the e13.5, e15.5, and e18.5 developmental stages. Interestingly, mouse ESCs were characterized by a very low level of 80 kDa coilin in comparison to embryonic brains (Figure 4(c)). In parallel with coilin, we analyzed fibrillarin levels in the mouse brains because individual CBs colocalize with fibrillarin in many cell types (Figures 4(c), 5(a), and 5(b)). By western blots, in mouse adult brains, we observed a very low level of fibrillarin (see two western blot expositions in Figure 4(c)), especially compared to mouse embryonic stem cells (mESCs). In our samples, shown in Figure 4(c), we found that when the level of coilin was high, the level of fibrillarin was low and vice versa.

Using western blot, we also detected the levels of 70 kDa coilin variant and 39 kDa fibrillarin in the OBs of the adult brain, the adult hippocampus, the brain cortex, and the whole adult brain (Figures 4(d) and 4(e)(B)). Compared to OBs, the hippocampus and the brain cortex were characterized by coilin depletion, which was accompanied by a decrease in HDAC1 level when it was normalized to total protein level and *α*-tubulin (Figures 4(d), 4(e)(B), and 4(e)(C)). The fibrillarin levels were not substantially different in the brain regions analyzed (Figure 4(d)).

Here, we also compared the nuclear pattern of CBs in mESCs and the human cancer cells line HeLa, which has been used by many authors for CBs studies [17, 33]. In HeLa cells, the CBs were always positive for both coilin and fibrillarin (Figure 5(a)). We additionally studied the nuclear distribution pattern of CBs and fibrillarin in nondifferentiated mESCs and mESCs undergoing neural differentiation (Figures 5(b) and 5(c)(A)–5(c)(D)). Wild-type and HDAC1 dn mESCs were characterized by a very subtle occurrence of fibrillarin in CBs (see white arrows in Figure 5(b)). However, in differentiated HDAC1 dn mESCs, robust foci of accumulated coilin appeared on the periphery of the nucleoli (Figure 5(c)(C); ~40% of cells) or high coilin positivity was found inside the nucleoli (Figure 5(c)(D); ~60% of cells). This nuclear distribution pattern of coilin was not observed in differentiated wt mESCs (Figures 5(c)(A) and 5(c)(B)).

**Figure 6:**
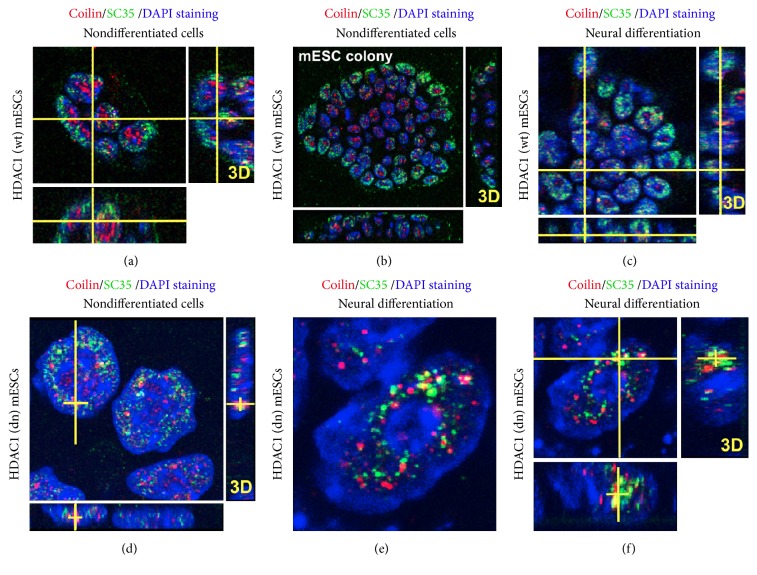
Spatial interactions between coilin and SC35-positive splicing speckles. (a) In wt mESCs, coilin (red) was distributed in the nuclear interior, and this coilin-positive region was surrounded by SC35 protein (green). (b, c) show that the mutual interaction between coilin and SC35 is changed during neural differentiation. Many cells were characterized by the formation of SC35-positive CBs (red). The colocalization tool in the Leica software showed ~30% colocalization between CBs (red) and SC35-positive nuclear speckles (green) in both (d) nondifferentiated HDAC1 dn mESCs and (e, f) differentiated HDAC1-depleted cells. A 3D projection (*x*-*y*-*z*) of interphase nuclei is documented in all panels.

Because CBs are nuclear regions associated with splicing processes, we additionally analyzed the spatial link between CBs and SC35-positive nuclear speckles (Figures 6(a)–6(f)). In mouse pluripotent ESC colonies, we observed high levels of coilin in the nuclear interior, and these regions were surrounded by the SC35 protein (Figures 6(a)–6(c)). We found that most of the CBs and SC35-positive nuclear speckles were spatially distinct, but some of them partially overlapped. This nuclear distribution pattern was identical in both wt and HDAC1 dn (Figures 6(a)–6(f)).

## 3. Discussion

CBs, which were first described by Cajal [1], consist of several proteins, including p80 coilin. The functional properties of coilin in CBs were characterized by Andrade et al. [34] and Raška et al. [12]. CBs are also the sites for various factors that play roles during pre-mRNA splicing, pre-ribosomal RNA processing, and histone pre-mRNA maturation [7, 33, 35]. Moreover, CBs are highly mobile structures, as demonstrated by photobleaching experiments [16, 17].

Here, we addressed the morphology of CBs in embryonic and adult brains and during the in vitro induction of mESC neural differentiation. Previously, in certain human and mouse ESCs (particularly at the periphery of mESC colonies), we observed the accumulation of coilin into visible CBs [10]. Conversely, human and mouse pluripotent ESCs, particularly those at the center of the colony, are highly positive for diffusely dispersed coilin protein (Figures 6(a) and 6(b); [10]). Thus, our results indicate that the peripheries of ESC colonies are more prone to spontaneous differentiation, which is characterized by an appearance of CBs [36]. Here, the formation of robust CBs or coilin-positive microfoci was more pronounced after induced neural differentiation, especially in HDAC1 dn ES cells (compare Figures 5(b) and 5(c)(A)–5(c)(D)). Our analyses confirmed that embryonic stem cells, characterized by an immense differentiation potential, are a good tool for the studies of nuclear architecture. For example, Butler et al. [37] showed the formation of CBs as a consequence of the spontaneous differentiation that frequently appears at the periphery of human ESC colonies, and it is also the case documented here. A good experimental model in which the formation of CBs is studied are the cells of the embryonic brain (particularly the cells in the prominent neurogenic regions destined for pronounced differentiation). For our analysis, we selected the hippocampus and the OBs (Figures 2(c)–2(f) and 3(b)(A)–3(b)(E)). Our data fit well with the original observations of Cajal, who noted the appearance of CBs in primary cells, such as the pyramidal cells from the human cerebral cortex (see also [8]) and the cells undergoing terminal differentiation (Figures 2(d), 3(b)(C), and 5(c)(A)–5(c)(D)). Here, for the first time, we show the accumulation of coilin in a crescent-like structure that is specific to the hippocampal regions of the adult brain (Figures 2(d) and 2(e)). Furthermore, OBs were characterized by well-visible CBs surrounded by high levels of coilin (Figures 3(b)(C)–3(b)(E)). These results support the conclusions of other authors that noted cell-type specificity regarding the size, morphology, and numbers of CBs [15, 38–44].

Here, we additionally revealed a link among the focal accumulation of coilin in the nucleolus, decreased coilin levels and HDAC1 depletion. This connection was particularly observed during the neural differentiation of mESCs and in the hippocampus (Figures 2(d), 2(e), 4(b), 4(d), 4(e)(A)–4(e)(C), 5(c)(C), and 5(c)(D)). In these cases, coilin was depleted and accumulated into robust CBs or microfoci inside the nucleoli of cells with an HDAC1 deficiency or HDAC1 decreased level. Thus, changes in histone acetylation, mediated by HDAC1 function, likely affected the interaction between coilin and chromatin-related factors. Accumulation of coilin to the nucleoli was found to be linked to coilin hypomethylation [5]. Interestingly, both HDAC1 depletion and coilin hypomethylation likely caused the coilin transition to the fibrillarin-positive dense fibrillar component of the nucleoli (compare Figures 4(b) and 4(e)(A) with Figures 5(b), 5(c)(C), and 5(c)(D) and [5]). Moreover, it seems to be possible that coilin could be hypomethylated in hyperacetylated surroundings in the genome, which can be caused by HDAC1 depletion. This epigenetic nuclear event could also be a consequence of HDAC1-dependent changes in chromatin accessibility.

## 4. Conclusion

Nuclear bodies, including CBs, are functionally important nuclear compartments containing accumulated proteins that play roles in many nuclear processes, including transcription, splicing, and DNA repair. The morphology and nuclear distribution patterns of these nuclear bodies likely reflect their functional properties, which contribute to the molecular mechanisms that maintain the balance between cell physiology and pathophysiology. We showed here that coilin is highly expressed in brain tissue, especially in the embryonic brain. Cajal bodies, recognized by accumulated coilin, were found to be localized inside nucleoli, especially in HDAC1-depleted cells, which was accompanied by coilin downregulation. These results show that epigenetic events, such as histone acetylation (or lysine pan-acetylation) affecting the accessibility of regulatory elements to chromatin, can stand behind changes in the nuclear distribution pattern of Cajal bodies.

## 5. Materials and Methods

### 5.1. Cell Cultivation

The nuclear distribution patterns of the coilin protein and its accumulation in CBs were analyzed in wt mESCs and HDAC1 dn mESCs (a generous gift from Dr. Christian Seiser, Max F. Perutz Laboratories, Vienna Biocenter, Austria) [32, 45]. Mouse ESCs were cultivated in DMEM (Thermo Fisher Scientific, USA) supplemented with 15% fetal bovine serum, 0.1 mM nonessential amino acids, 100 *μ*M MTG, 1 ng/mL leukemia inhibitory factor (LIF), 10,000 IU/mL penicillin, and 10,000 *μ*g/mL streptomycin. Culture dishes were coated with Matrigel (#354277, Corning, USA) according to the protocols described by Franek et al. [46]. Neural differentiation was induced in medium without LIF. After two days, the medium was replaced with serum-free commercial DMEM/F-12 (1 : 1) (GIBCO, UK) supplemented with insulin, transferrin, and selenium (ITS-100x, GIBCO, UK), 1 *μ*g/mL fibronectin (Sigma-Aldrich, Czech Republic), and penicillin/streptomycin (according to Pacherník et al. [47] describing this DMEM/F-12/ITSF medium). In the next two days, this medium was additionally supplemented by 0.5 *μ*M* all-trans* retinoic acid (ATRA, Sigma-Aldrich, Czech Republic) that was replaced at day 4 by DMEM/F-12/ITSF medium.

HeLa-Fucci cells were purchased and cultivated as previously described [48].

### 5.2. Tissue Sectioning and Immunostaining

Adult and embryonic mouse brains (at developmental stages e13.5, e15.5, and e18.5 after fertilization; mouse strain C57Bl6) were maintained in tissue freezing medium (OCT embedding matrix, Leica Microsystems, Germany) at −20°C. A Leica cryomicrotome (Leica CM 1800, Leica, Germany) was used for tissue sectioning. Tissue sections were washed in PBS and postfixed in 4% formaldehyde for 20 min for immunostaining. The tissues were permeabilized in 1% Triton X-100 and 0.1% saponin (Sigma-Aldrich, Czech Republic) dissolved in PBS. Immunohistochemistry was performed according to the protocols described by Bártová et al. [49]. In our studies, we used a primary antibody raised against coilin (H-300) (#sc-32860, Santa Cruz, USA), fibrillarin (#ab4566, Abcam, UK), and a goat anti-rabbit Alexa Fluor 594 secondary antibody (#A11012, Invitrogen) or goat anti-mouse Alexa Fluor 594 (#A11032, Invitrogen, USA) or anti-rabbit Alexa Fluor 488 (#ab150077, Abcam, UK). The primary antibodies were diluted 1 : 100, and the secondary antibodies were diluted 1 : 200 in PBS containing 1% BSA. The DNA was counterstained with DAPI (4′,6-diamidino-2-phenylindole) (Sigma-Aldrich, branch in the Czech Republic) dissolved in the mounting medium Vectashield (Vector Laboratories, USA).

We additionally used an antibody raised against acetylated H3K9 (#06-942, Merck Millipore, Czech Republic) to visualize the granular layer of the OBs (Figure 3(a)(A)).

### 5.3. Western Blots

Western blot was performed according to the protocols described by Krejčí et al. [50]. To analyze coilin levels by western blot, we used an antibody raised against coilin (#sc-32860, Santa Cruz, USA) at a dilution of 1 : 1000. Coilin levels were analyzed in nondifferentiated and differentiated mESCs as well as embryonic and adult brains. In addition, we examined fibrillarin, histone deacetylase 1 (HDAC1), pan-acetylated lysine, and *α*-tubulin levels using the following antibodies: fibrillarin (#ab5821, Abcam, UK), HDAC1 (#sc7872, Santa Cruz Biotechnology, Inc., USA), anti-pan-acetylated lysine (#ab21623, Abcam, UK), and *α*-tubulin (#LF-PA0146, Thermo Fisher Scientific Inc., branch in Czech Republic). The secondary antibody was a peroxidase-conjugated anti-rabbit IgG (#A-4914; Sigma, Munich, Germany) diluted 1 : 2000. Equal amounts of protein were loaded in each gel lane. Protein levels were normalized to the total protein levels measured with a *μ*Quant spectrophotometer and the KCjunior software (BioTek Instruments, Inc., Winooski, VT, USA) or to total histone H3 levels (#ab1791, Abcam, UK).

### 5.4. Confocal Microscopy and Image Analysis

We acquired images with a Leica TCS SP5 X confocal microscope (Leica Microsystems, Germany). Image acquisition was performed using a white light laser (WLL) with the following parameters: 1024 × 1024-pixel resolution, 400 Hz, bidirectional mode, and zoom 8–12. For 3D projections, we obtained 30–40 optical sections with axial steps of 0.3 *μ*m. 3D projection reconstruction was conducted using the Leica Application Suite (LAS) software. The scanning of larger biological objects, such as embryonic brain sections, was conducted in tile scanning mode with the Leica software, as previously described [49].

### 5.5. Statistical Analysis

We used Excel software for data presentation. Florescence intensity and density of western blot fragments were calculated by ImageJ software (NIH freeware). Statistically significant results at *p* ≤ 0.05 (*p* ≤ 0.01) are labeled by asterisks *∗* (*∗∗*). Statistical analysis was performed by Student's *t*-test, a tool of Sigma Plot 8.0 software.
